# Influence of host cell line and microsporidian species in the *in vitro* infection efficiency of *Encephalitozoon* spp.

**DOI:** 10.1051/parasite/2026026

**Published:** 2026-04-23

**Authors:** Leslie Vercruysse, Valentine Daugey, Aurore Dubuffet, Céline Lambert, Mathilde Bonnet, Virginie Bonnin, Philippe Poirier, Céline Nourrisson

**Affiliations:** 1 Université Clermont Auvergne, INSERM UMR 1071, INRAE USC 1382, “Microbes, Intestin, Inflammation et Susceptibilité de l’Hôte” M2iSH F-63000 Clermont-Ferrand France; 2 Université Clermont Auvergne, CNRS, “Laboratoire Microorganismes : Génome et Environnement” LMGE 1 impasse Amélie Murat - 63170 Aubière France; 3 Unité de Biostatistiques, DRCI, CHU Clermont-Ferrand 58 rue Montalembert 63000 Clermont-Ferrand France; 4 Université Clermont Auvergne, CHU Clermont-Ferrand, INSERM UMR 1071, INRAE USC 1382, Service de Parasitologie-Mycologie, “Microbes, Intestin, Inflammation et Susceptibilité de l’Hôte” M2iSH 58 rue Montalembert 63000 Clermont-Ferrand France

**Keywords:** Microsporidia, *Encephalitozoon*, Cell culture, *In vitro* models, Infection rate, Infectious foci

## Abstract

Microsporidia are obligate intracellular eukaryotes infecting a wide range of hosts and cell types. The development of *in vitro* culture models is essential for studying host–parasite interactions and the pathogenesis of microsporidian infections. In this study, we compared the infection efficiency of three human pathogenic species of the genus *Encephalitozoon* (*Encephalitozoon intestinalis*, *E. hellem*, and *E. cuniculi*) across six cell lines: TC7, HT-29, HCT 116, T84, Vero, and MRC-5. Infection rates and the surface area of parasitic foci were determined after labeling microsporidia by fluorescence *in situ* hybridization (FISH). Both parameters varied significantly according to the cell line and *Encephalitozoon* species. TC7 cells consistently supported the highest infection rates, whereas HCT 116 cells were the least permissive. The surface areas of foci were primarily species-dependent, with larger foci observed for *E. cuniculi* and smaller ones for *E. intestinalis*. In conclusion, these results revealed marked differences in invasive and proliferative dynamics depending on the *Encephalitozoon* species and cell line and highlight the crucial impact of cell line selection on *in vitro* experimental outcomes. This work provides a foundation for improving and standardizing experimental models for future experiments on *Encephalitozoon* spp.

## Abstract (fr)

Les microsporidies sont des eucaryotes intracellulaires obligatoires qui infectent un large éventail d’hôtes et de types cellulaires. Le développement de modèles de culture *in vitro* est essentiel pour étudier les interactions hôte-parasite et la pathogenèse des infections microsporidiennes. Dans cette étude, nous avons comparé l’efficacité d’infection de trois espèces pathogènes pour l’Homme du genre *Encephalitozoon* (*Encephalitozoon intestinalis*, *E. hellem* et *E. cuniculi*) sur les six lignées cellulaires suivantes : TC7, HT-29, HCT 116, T84, Vero et MRC-5. Les taux d’infection et l’aire des foyers parasitaires ont été déterminés après marquage des microsporidies par hybridation *in situ* en fluorescence (FISH). Les deux paramètres variaient significativement selon la lignée cellulaire et l’espèce d’*Encephalitozoon*. Les cellules TC7 présentaient systématiquement les taux d’infection les plus élevés, tandis que les cellules HCT 116 étaient les moins permissives. L’aire des foyers était principalement dépendante de l’espèce, avec des foyers généralement plus grands pour *E. cuniculi* et plus petits pour *E. intestinalis*. En conclusion, ces résultats révèlent une variabilité considérable des dynamiques invasives et prolifératives selon l’espèce d’*Encephalitozoon* et la lignée cellulaire, et soulignent l’impact crucial du choix de la lignée cellulaire sur les résultats expérimentaux *in vitro*. Ce travail fournit une base pour améliorer et standardiser les modèles expérimentaux pour les futures expériences sur *Encephalitozoon* spp.

## Introduction

Microsporidia are obligate intracellular eukaryotes related to Fungi and capable of infecting a wide range of animal species [11]. Of the 1,700 species of microsporidia classified into 220 genera, 17 have been identified as pathogenic to humans to date [7]. Among these, *Enterocytozoon bieneusi* is by far the most frequently detected [11]. However, this species remains poorly studied due to the current inability to establish it in continuous *in vitro* culture [34]. *Encephalitozoon* is the second most frequently implicated genus in human infections and includes three species that are pathogenic to humans: *Encephalitozoon intestinalis*, *E. cuniculi*, and *E. hellem* [11]. Unlike *E. bieneusi*, these three species of *Encephalitozoon* can be cultured *in vitro*, allowing for more in-depth study of their life cycle and pathophysiological mechanisms [11, 23]. Numerous *in vitro* studies have investigated various aspects of *Encephalitozoon* spp. biology, including mechanisms of host cell invasion, life cycle progression, and host cell responses [25, 34]. However, there is considerable variability between these studies in terms of experimental models, such as the choice of cell lines and microsporidia species, as well as in spore preparation protocols, and the duration of experimental infection and its follow-up. This heterogeneity complicates comparative analyses and synthesis of results from the literature [10].

*Encephalitozoon* spp. are characterized in particular by: (i) a life cycle of 48–72 h divided into three distinct phases: host cell invasion, intracellular proliferation, and spore formation, and (ii) intracellular development within a parasitophorous vacuole forming a cluster of microsporidia, also called an infectious focus [13]. The spore, which represents the infectious stage, contains the sporoplasm (*i.e.*, the cytoplasm, nucleus, and organelles) which is transferred into the host cell through a filamentous structure called the polar tube [13]. Thus, during *in vitro* culture, the multiplication of microsporidia in cells leads to the release of immature stages and mature spores into the culture supernatant. The cell lines most commonly used for such production of *Encephalitozoon* spp. spores in continuous *in vitro* cultures are rabbit kidney cells (RK13 cells) and African green monkey kidney cells (Vero cells) [22, 31]. Methods for purifying spores released into the culture supernatant range from simple filtration [22] to more sophisticated protocols using Percoll density gradient centrifugation [1, 33] in order to separate infectious spores from non-infectious spores and immature stages. Finally, the cell models used for experimental infections are diverse and include Vero cells [1], human colonic carcinoma cells (Caco-2, HCT-8, HT-29, SW480 cells) [20], human macrophages (THP-1, RAW 264.7 cells) [27, 31], and fibroblasts (HFF, MRC-5 cells) [11].

The proportion of cells infected during an experiment will be a major parameter that will influence the detection of molecular disturbances in host cells within the cell monolayer (containing infected and uninfected cells). This proportion should however be high enough to reach satisfactory statistical power to measure biological effects, such as the effect of infection on a biological parameter or the effects of a treatment on the proportion of infected cells. Importantly, when only a small fraction of host cells is infected, treatment-related differences are masked by the dominant uninfected background, so observed effect sizes are small and often fail to reach statistical significance.

Although the first *in vitro* studies on *Encephalitozoon* date back to 1969 [30], very little data are currently available on the infection rates of different *Encephalitozoon* species and cell lines. To date, only one study, published in 2006, has evaluated the variability of *in vitro* infection efficiency between different strains of *E. hellem* in Vero E6 cells [14]. However, the study focused on the number of infectious foci (*i.e.*, clusters of spores) within the cells and not on the percentage of infected cells.

Consequently, it remains unclear which cell lines maximize the proportion of infected cells for studying *Encephalitozoon* spp. The aim of the present study was to compare the infection efficiency of strains of *E. intestinalis*, *E. cuniculi*, and *E. hellem* in six different cell lines previously used for the culture of *Encephalitozoon* sp.: TC7, HT-29, HCT 116, T84, Vero, and MRC-5 cells, by measuring infection rates and the surface area of infectious foci.

## Material and methods

### Culture of the different cell lines

HT-29 cells (ATCC HTB-38) and HCT 116 cells (ATCC CCL-247) are derived from human adenocarcinoma and colorectal carcinoma, and exhibit an epithelial morphology. Both cell lines were cultured at 37 °C, under 5% CO_2_ in McCoy’s 5A medium (Gibco, Thermo Fisher Scientific, Waltham, MA, USA) supplemented with 10% fetal bovine serum (Dutscher, Bernolsheim, France), 100 U/mL penicillin, 0.1 mg/mL streptomycin, and 0.25 μg/mL amphotericin B (Cytiva, Marlborough, MA, USA).

Caco-2/TC7 cells (ATCC HTB-37), derived from human colorectal adenocarcinoma, have an epithelial-like morphology. The TC7 clone was isolated from a late passage of the parental Caco-2 line. These cells were cultured at 37 °C, under 5% CO_2_ in DMEM (Dulbecco’s Modified Eagle Medium) high glucose (Gibco) supplemented with 10% fetal bovine serum (Dutscher), 100 U/mL penicillin, 0.1 mg/mL streptomycin, 0.25 μg/mL amphotericin B (Cytiva), 1× vitamin MEM L-Glutamine free (Dutscher), and 1× non-essential amino acids MEM (Minimum Essential Medium, Gibco).

Vero cells (ATCC CCL-81) are derived from African green monkey kidney and have an epithelial morphology. MRC-5 cells (ATCC CCL-171), derived from human lung, have a fibroblastic morphology. Both cell lines were cultured at 37 °C, under 5% CO_2_ in MEM (Gibco) supplemented with 10% fetal bovine serum (Dutscher), 100 U/mL penicillin, 0.1 mg/mL streptomycin, and 0.25 μg/mL amphotericin B (Cytiva).

T84 cells (ATCC CCL-248) are derived from a lung metastasis of a human colorectal carcinoma and exhibit an epithelial morphology. These cells were cultured at 37 °C, under 5% CO_2_ in DMEM F12 (Gibco) supplemented with 10% fetal bovine serum (Dutscher), 100 U/mL penicillin, 0.1 mg/mL streptomycin, 0.25 μg/mL amphotericin B (Cytiva), 1× vitamin MEM L-Glutamine free (Dutscher), and 10 mM Hepes buffer (Biowest, Nuaillé, France).

All cell lines were passaged twice a week. Mycoplasmas were tested for once per quarter in all cell lines by PCR.

### Culture of *Encephalitozoon intestinalis*, *E. cuniculi*, and *E. hellem*

The microsporidia *E. cuniculi* (GB-M, genotype I), *E. intestinalis* (ATCC 50506), and *E. hellem* (isolated from patient stool and previously axenised in our laboratory [28], genotype 2B) were cultured on rabbit kidney RK13 cells (ATCC CCL-37) at 37 °C, under 5% CO_2_ in MEM (Gibco) supplemented with 5% fetal bovine serum (Dutscher), 100 U/mL penicillin, 0.1 mg/mL streptomycin, and 0.25 μg/mL amphotericin B (Cytiva). The medium was replaced twice a week, and spores released into the culture supernatant were harvested at each medium change.

### Purification of the collected spores

The culture supernatants of *E. intestinalis, E. cuniculi*, and *E. hellem* were collected and centrifuged at 4 °C for 10 min at 1,300× *g*. The pellet was then resuspended in 30 mL of sterile distilled water. To lyse any remaining RK13 cells, the suspension was passed once through a 27 G needle and subsequently centrifuged at 4 °C for 10 min at 1,300× *g*. The resulting pellet was washed three times with 1× PBS. Spores in the pellet were stained with Calcofluor White (Sigma-Aldrich, St. Louis, MO, USA) and counted in a Kova cell. Fluorescence (ex/em: 356/451 nm) was observed with a 40× objective on a ZEISS Axio Observer microscope (Carl Zeiss Microscopy, Jena, Germany).

### Infection of the different cell lines by *Encephalitozoon intestinalis*, *E. cuniculi*, and *E. hellem*

Cell lines were seeded at a density of 5 × 10^4^ cells/cm^2^ (10^5^ cells/well) for HCT 116, TC7, MRC-5, and Vero cells, or at 8 × 10^4^ cells/cm^2^ (1.5 × 10^5^ cells/well) for HT-29 and T84 cells, in 24-well plates (Falcon) containing glass coverslips.

Twenty-four hours after seeding, cleaned spores of the three *Encephalitozoon* species were added to infect the cells at a multiplicity of infection of 100 spores per cell. The spores were incubated with the cells for three hours, after which the wells were washed three times with 1× PBS, and fresh culture medium was added. Forty-eight hours post-infection (PI), the medium was removed and the cells were fixed by addition of 300 μL of methanol per well. The plates were then stored at −20 °C.

Each condition was tested in triplicate, and each experiment was repeated three times independently.

### Labeling of *Encephalitozoon intestinalis*, *E. cuniculi*, and *E. hellem*

Infected cells were detected by fluorescence *in situ* hybridization (FISH). Briefly, after rehydration with 1× PBS for 10 min, the cells underwent a pre-hybridization step, gradually replacing the PBS with a hybridization buffer (HB) (20 mM Tris-HCl pH 7.8; 0.9 M NaCl; 1× Denhardt’s solution (Invitrogen, Thermo Fisher Scientific); 0.01% SDS) during successive 10-minute incubations at room temperature with PBS: HB (volume to volume), followed by HB alone, and finally 15 min incubation at 48 °C with HB. The coverslips were then incubated with probes targeting ribosomal RNAs: “INT1” (5′–Cy3-GTTCTCCTGCCCGCTTCAG–3′) for *E. intestinalis* [9], or “HEL878F” (5′–Cy3-ACTCTCACACTCACTTCAG–3′) for *E. hellem* [16], or “Ec01” (5′–Cy3- CCACAGGGGCAGACCACTAT–3′) for *E. cuniculi* [5] at the concentration of 0.5 μM for 3 h at 48 °C in a hybridization incubator (Slide Moat, Boekel, Feasterville, PA, USA). After successive washes with HB at 48 °C for 20 min and at room temperature for 5 min, followed by a PBS: HB mixture wash at room temperature for 10 min, DAPI labeling (300 nM, Cell Signaling Technology) was performed. Coverslips were then mounted with ProLong Diamond Antifade Mountant (Invitrogen) and observed using a ZEISS Axio Observer microscope (Carl Zeiss Microscopy) with a 40× objective. Host cell nuclei were counted using DAPI fluorescence (ex/em: 350/470 nm), while the number of infected cells was determined with the fluorescence emitted from the Cy-3-labelled FISH probes (ex/em: 554/566 nm). For each cell line and each species of *Encephalitozoon*, 1,000 cells from the cellular monolayer were observed in each of the three separate glass coverslips for each independent biological replicate (*i.e.*, a total of nine coverslips).

### Total cell count

For each cell line, additional wells were seeded to calculate the total cell count 24 h post-seeding and 48 h PI. Briefly, at both times, the cells were washed with 1× PBS, trypsinized, and harvested for counting using the ADAM-MC cell counter (NanoEntek, Seoul, South Korea), according to the manufacturer’s instructions. Briefly, this automated fluorescence cell counter performs cell viability and count measurements using the propidium iodide dead-cell staining method combined with advanced image analysis.

### Calculation of the infection rate

The total number of infected cells at 48 h PI was calculated by multiplying the percentage of infection (*i.e.*, [number of cells containing infectious foci / total number of cells counted] × 100) by the total number of cells at 48 h PI, as determined by the cell counter (without considering cell viability). The infection rate was then obtained by dividing the total number of infected cells at 48 h PI by the number of viable cells at the time of infection (*i.e.*, 24 h after seeding in our protocol) and multiplying by 100. Thus the calculation was as follows:

$$\begin{array}{c} \text{Infection rate}\left(\%\right)\left(\frac{\text{percentage of infected cells at}48\text{hours PI}\times \text{total number of cells at}48\text{hours PI}}{\text{number of cells on the day of infection}}\right)\times 100. \end{array}$$

This calculation allows for the exclusion of: (i) seeded cells that did not adhere to the well, and (ii) the proliferation of uninfected cells during the 48 h following infection. For each cell line and each species of *Encephalitozoon*, 1,000 cells from the cellular monolayer were observed in each of the three separate glass coverslips for each independent biological replicate (*i.e.*, a total of nine coverslips). The number of infected cells was counted among these 1,000 cells.

### Measurement of infectious foci areas

The surface area of the infectious foci labeled by FISH was measured using QuPath software (https://qupath.github.io/) [3]. For each cell line and each species of *Encephalitozoon*, 1,000 cells were observed in three separate glass coverslips for each independent biological replicate (*i.e.*, nine coverslips). Among these 1,000 cells, all foci of each infected cell were measured.

### Statistical analyses

Statistical analyses were performed using Stata software (version 16; StataCorp, College Station, TX, USA). All tests were two-sided, with a significance level set at 0.05. Infection rates and surface areas of the infectious foci are reported as mean ± standard deviation and/or median [25th; 75th percentiles]. Comparison of infection rates among the three *Encephalitozoon* species was performed using linear regression, adjusting for the cell line. Comparison of infection rates among the six cell lines was performed separately for each species using the Kruskal–Wallis test, followed by Dunn’s *post hoc* multiple comparisons test. Comparison of surface areas of the infectious foci among the three *Encephalitozoon* species was performed using a linear mixed model with log-transformed surface areas, adjusting for cell line, and with biological replicate as a random effect. Comparison of surface areas of the infectious foci among the six cell lines was performed separately for each species using a linear mixed model with log-transformed surface areas, and biological replicate as a random effect. All *post hoc* pairwise comparisons were performed when the omnibus *p*-value was ≤ 0.05, with Šidàk correction.

## Results

### Comparison of the three *Encephalitozoon* spp.

Regardless of cell line, the average infection rates were significantly different among the three species of *Encephalitozoon* (*p* ≤ 0.001) and reached 14.92 ± 9.99% for *E. intestinalis*, 38.58 ± 29.12% for *E. hellem*, and 45.10 ± 30.47% for *E. cuniculi*. *Post hoc* pairwise comparisons revealed a significant difference between *E. intestinalis* and *E. hellem* on the one hand (*p* = 0.001), and between *E. intestinalis* and *E. cuniculi* on the other (*p* < 0.001).

Regardless of cell line, the surface areas of the infectious foci were significantly different among the three species (*p* ≤ 0.001): 45 [24; 86] μm^2^ for *E. intestinalis*, 64 [34; 119] μm^2^ for *E. hellem*, and 110 [64; 186] μm^2^ for *E. cuniculi*. All *post hoc* pairwise comparisons were statistically significant (*p* ≤ 0.001).

### Infection by *Encephalitozoon intestinalis*

**Figure 1:**
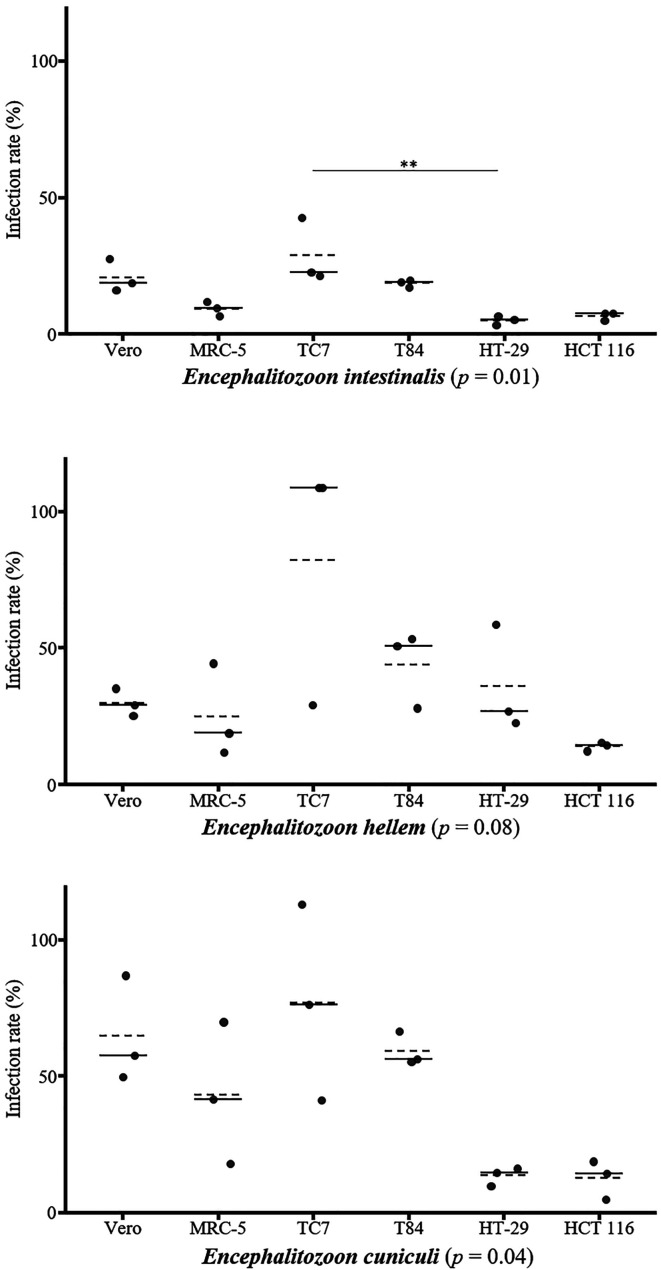
Infection rates of three species of *Encephalitozoon* in six cell lines. Quantification of cells infected by *E. intestinalis*, *E. hellem*, and *E. cuniculi* at 48 hours post-infection in six different cell lines. The results were obtained from three biological replicates with *n* > 1,000 cells per experiment. The mean is represented by a dashed line and the median by a solid line. The overall *p*-value is shown in parentheses for each species. *Post hoc* pairwise comparisons are indicated by asterisks: *p ≤ 0.05, **p ≤ 0.01.

The average infection rate by *E. intestinalis* varied depending on the cell line and can be ranked in ascending order, from least infected to most infected, as follows: HT-29 (5.12 ± 1.57%), HCT 116 (6.66 ± 1.54%), MRC-5 (9.36 ± 2.62%), T84 (18.64 ± 1.29%), Vero (20.86 ± 5.99%), and TC7 (28.86 ± 11.87%) (Fig. 1, Table S1). There was a statistically significant difference in infection rates among cell lines (*p* = 0.01). *Post hoc* pairwise comparisons revealed a significant difference only between TC7 and HT-29 cells (*p* = 0.01) (Table S2).

**Figure 2:**
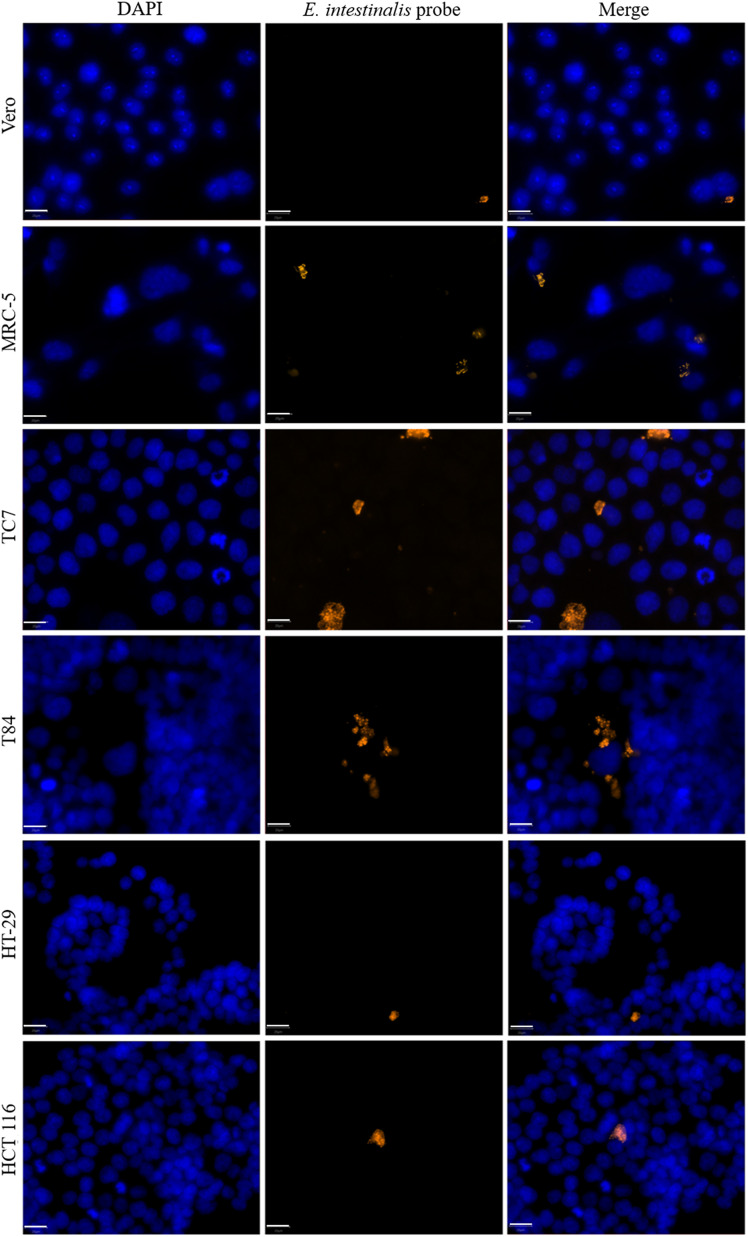
*Encephalitozoon intestinalis* infectious foci in six different cell lines. Microscopic detection of *E. intestinalis* in six different cell lines 48 hours post-infection by FISH (blue: DAPI; orange: FISH-labeled *E. intestinalis* foci). Scale bar: 20 μm.

**Figure 3:**
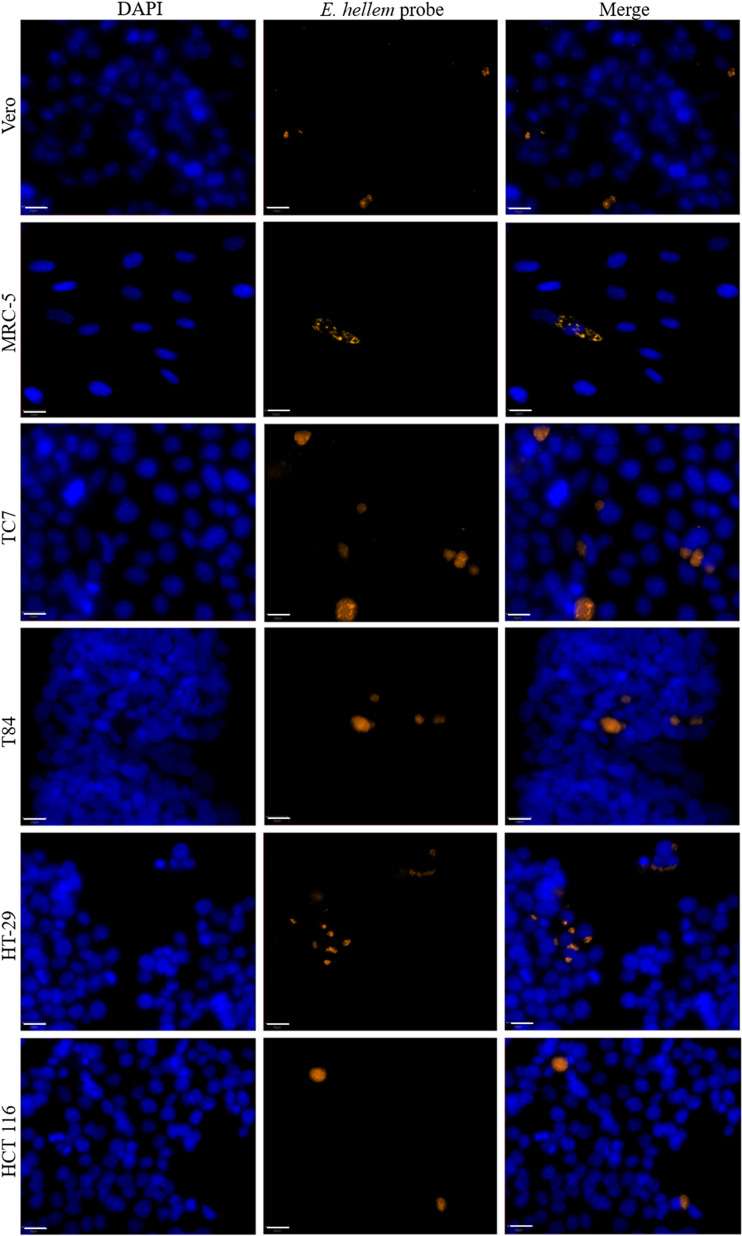
Surface areas of three species of *Encephalitozoon* in six cell lines. Measurement of the surface area of infectious foci within cells infected by *E. intestinalis*, *E. hellem*, and *E. cuniculi* at 48 h post-infection. The results were obtained from three biological replicates. The number of areas measured is indicated under each cell line. The median is represented by a solid line. The overall *p*-value is shown in parentheses for each species. *Post hoc* pairwise comparisons are indicated by asterisks: *p ≤ 0.05, **p ≤ 0.01, ***p ≤ 0.001.

The median surface area of *E. intestinalis* infectious foci varied depending on the cell line (Fig. 2). The foci were significantly smaller in Vero cells (25 [14; 37] μm^2^) compared to HT-29, T84, MRC-5, and TC7 cells (45 [26; 70], 52 [24; 86], 70 [33; 98], and 77 [35; 134] μm^2^, respectively), which were the three cell lines containing the largest foci (Fig. 3A, Table S3). For HCT 116 cells, the median surface area of the foci was 35 [16; 66] μm^2^.

### Infection by *Encephalitozoon hellem*

The average infection rate by *E. hellem* varied depending on the cell line and can be classified in ascending order as follows: HCT 116 (14.12 ± 1.62%), MRC-5 (25.09 ± 17.13%), Vero (29.91 ± 5.10%), HT-29 (36.06 ± 19.59%), T84 (44.07 ± 13.95%), and TC7 (82.21 ± 45.98%) (Fig. 1, Table S1). There was no statistically significant difference in infection rates among cell lines (*p* = 0.08).

**Figure 4:**
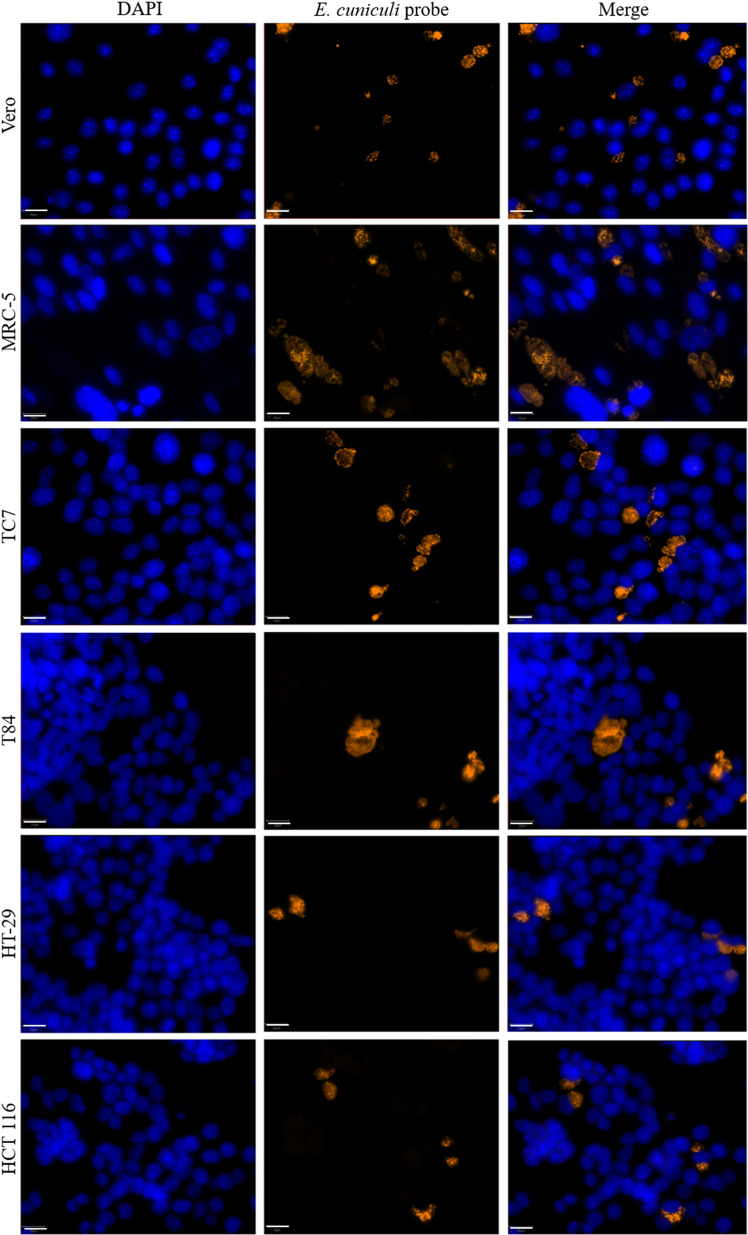
*Encephalitozoon hellem* infectious foci in six different cell lines. Microscopic detection of *E. hellem* in six different cell lines 48 hours post-infection by FISH (blue: DAPI; orange: FISH-labeled *E. hellem* foci). Scale bar: 20 μm.

The median surface area of *E. hellem* infectious foci varied depending on the cell line (Fig. 4). The foci were significantly smaller in HT-29 cells (43 [26; 70] μm^2^) than in the other five cell lines, *i.e.*, TC7, HCT 116, MRC-5, T84, and Vero cells (67 [37; 134], 78 [35; 134], 80 [43; 169], 100 [52; 187], and 115 [65; 185] μm^2^, respectively) (Fig. 3B, Table S3).

### Infection by *Encephalitozoon cuniculi*

The average infection rate by *E. cuniculi* varied depending on the cell line. The classification of infection rates from lowest to highest shows that HCT 116 cells were the least infected (12.76 ± 7.03%) followed by HT-29 (13.61 ± 3.28%), MRC-5 (43.20 ± 25.90%), T84 (59.32 ± 6.07%), Vero (64.81 ± 19.54%), and TC7 (76.88 ± 35.77%) (Fig. 1, Table S1). There was a statistically significant difference in infection rates among cell lines (*p* = 0.04), but no significant differences were found in *post hoc* pairwise comparisons (Table S2).

**Figure 5:**
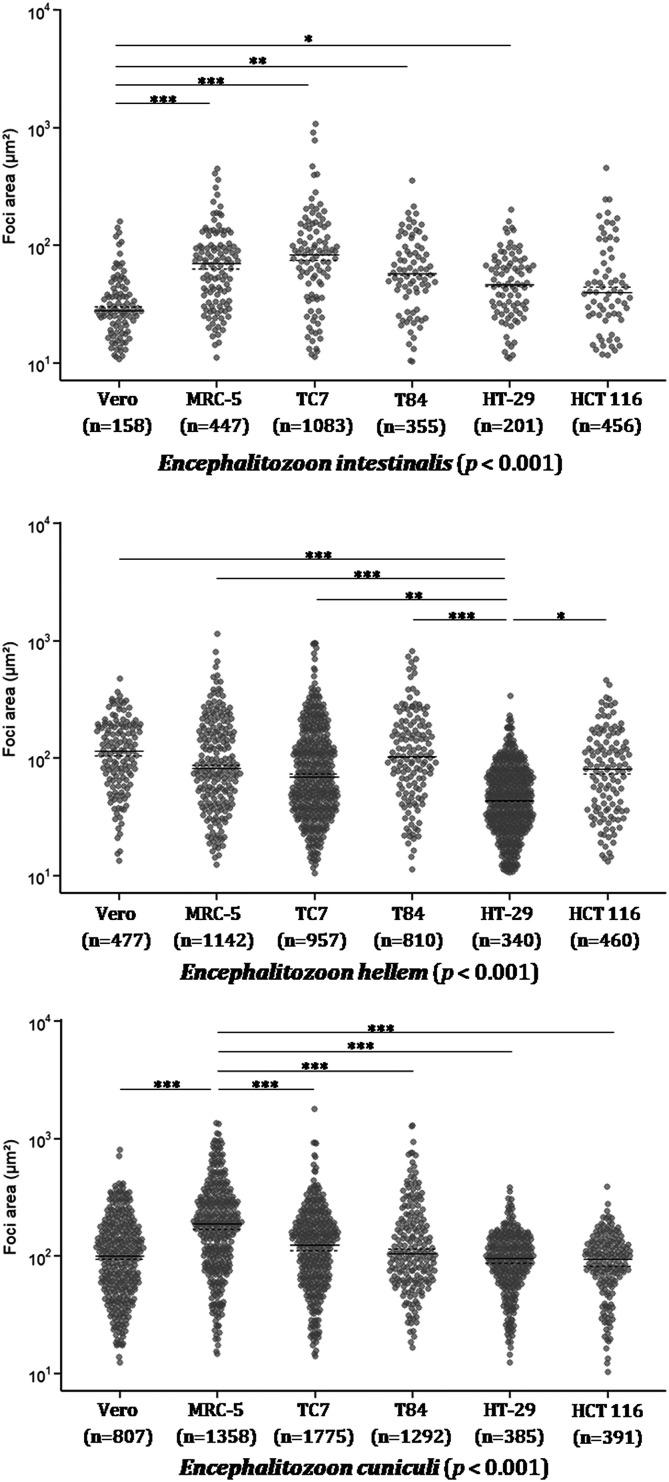
*Encephalitozoon cuniculi* infectious foci in six different cell lines. Microscopic detection of *E. cuniculi* in six different cell lines 48 hours post-infection by FISH (blue: DAPI; orange: FISH-labeled *E. cuniculi* foci). Scale bar: 20 μm.

The median surface area of *E. cuniculi* infectious foci varied depending on the cell line (Fig. 5). The foci were significantly larger in MRC-5 cells (187 [90; 304] μm^2^) than in the other five cell lines (Fig. 3C, Table S3). The median surface areas of the other cell lines were as follows, in ascending order: 94 [62; 128] μm^2^ for HCT 116 cells, 95 [62; 133] μm^2^ for HT-29 cells, 100 [52; 167] μm^2^ for Vero cells, 105 [62; 198] μm^2^ for T84 cells, and 125 [65; 192] μm^2^ for TC7 cells.

Discussion

### Choice of cell model

Successful infection of a cultured microorganism is a prerequisite for obtaining reliable *in vitro* results. The selection of an appropriate cell model depends on several factors, including: (i) its relevance to the scientific question – for example, HT-29 cells are particularly useful for the study of human colorectal cancer, while Vero cells are more commonly used for studies on pathogens, and (ii) its susceptibility to the pathogen of interest. In this study, intestinal cell lines were preferred due to typical tropism of *E. intestinalis* and *E. hellem* for the gut. However, other cell lines known for their high susceptibility to microsporidia, such as Vero epithelial cells [1] and MRC-5 fibroblasts [4], were also included in the analysis. Furthermore, intrinsic characteristics of the cell line, such as the proliferation rate, may influence its selection in the first instance and the experimental protocol in the second instance, as fast-growing lines are often easier to control experimentally. For example, in these experiments, HCT 116 and Vero cells proliferated faster than HT-29 and T84 cells, which justifies the different cell seeding used in our protocol.

### Variability in proliferative dynamics depending on the *Encephalitozoon* species and cell line

Using indirect approaches, such as measurements of infection rate and foci area, the present study revealed considerable variability in invasive rates and proliferative dynamics depending on the *Encephalitozoon* species and cell line. This observation is consistent with the findings of Haro *et al.* [14], who focused specifically on the ‘strain’ effect within a single species by studying the proliferation kinetics in Vero E6 cells of four strains of *E. hellem* with different polar tube protein genotypes. They used Gram-Chromotrope staining and calculated an infection foci score after prolonged incubation periods (9, 16, 20, and 24 days PI). They observed different proliferative dynamics depending on the strain of *E. hellem*. In our study, we demonstrated that this variability also depends on the cell line used. Regardless of the *Encephalitozoon* species, TC7 cells exhibited higher infection rates, while HCT 116 cells were the least infected. These different susceptibilities of cells to invasion may result from various cell-dependent factors, such as membrane composition, including the expression of specific receptors and glycosylation, or differences in cell differentiation status, which may influence parasite adhesion and internalization [20]. For example, it has been shown that transferrin receptor 1 plays a major role in the invasion by *E. hellem* [12] and that the attachment of *E. intestinalis* spores to the apical surface was greater on undifferentiated Caco-2 cells than on differentiated cells and greater on partially differentiated HCT-8 cells than on differentiated HCT-8 cells [20]. While the susceptibility of TC7 cells to *Encephalitozoon* spp. requires further confirmation in additional studies (for example by extending the experimental duration to study whether this cell line can produce infectious spores), these cells represent a potentially valuable model. It is worth noting that among the digestive tract cells tested, TC7 cells are the only ones to have a phenotype closer to the small intestine than to the colon, which is consistent with the preferential tropism of *Encephalitozoon*. However, their use may be subject to inter-experimental variability, as shown by the standard deviations obtained with each of the three species despite uniform cultivation conditions. Interestingly, if we compare the three species, infection rates for *E. intestinalis* were generally lower and those of *E. cuniculi* higher, regardless of the cell line.

With regard to the foci area, and therefore indirectly to the proliferative phase, the effect observed seems to be more related to the microsporidia species than to the cell line. One might have assumed, however, that it was the cell’s ability to withstand “stretching” of its cytoplasm that was the limiting factor in the spread of foci before the cell finally bursts. However, if we compare the three species, firstly, it was not the same cell lines that contained the largest foci, and secondly, the foci of *E. intestinalis* were generally smaller and those of *E. cuniculi* larger, regardless of the cell line. Larger foci could indicate more efficient intracellular replication, better adaptation to the host’s metabolism, or a reduced ability of the host cell to control the infection. These results therefore pave the way for further investigations.

Overall, there appeared to be no correlation between invasion and proliferation; however, TC7 cells appeared to be associated with greater infection efficiency. This issue of differences in infection efficiency depending on cell line has been described for other intracellular pathogens. For example, numerous studies have been published on the protozoan *Trypanosoma cruzi* [8] and several factors have been shown to impact infection dynamics, such as presence of serum, temperature, and type of host cell. The same problem is also encountered with viruses. For example, one study compared the replication kinetics and infectivity of several SARS-CoV-2 variants on three distinct cell models and showed clear differences depending on the cell line used, but also on the viral variant [24]. In the present study as well, infection efficiency was linked to the ‘variant’ of the pathogen used, here the *Encephalitozoon* species. Among the three species tested, *E. cuniculi* was associated with higher infection rates and larger foci than the other two species, particularly *E. intestinalis*. To our knowledge, there is no formal explanation for this phenomenon. However, it is worth noting that *E. cuniculi* is associated with systemic infections, unlike *E. intestinalis*, which is more restricted to the intestinal tract. This correlates well with the greater ease of culturing *E. cuniculi* on different cell lines and probably explains why *E. cuniculi* is the species most commonly used for *in vitro* studies [34]. It is interesting to note that this species was the first of the genus *Encephalitozoon* to be continuously cultured *in vitro* in 1969 [30] and that, for the following 20 years, it was the only mammalian microsporidia cultured *in vitro* [17]. Therefore, it cannot be ruled out that the *E. cuniculi* strains commonly used in the laboratory have long been “adapted” to *in vitro* culture conditions, which could explain their higher infection rates. This phenomenon has already been well described with *Plasmodium falciparum*, whose new clinical isolates exhibit replication rates lower than those of the long-term laboratory adapted clones [26].

### Areas for improvement

Recently, a new protocol purifying *E. intestinalis* spores using Percoll^®^ gradients was published, enabling infection rates of up to 80% on Vero cells with a multiplicity of infection of 30 spores per seeded cell [2]. This protocol is based on the exclusive collection of infectious spores, in contrast to ours, which recovers all microsporidian stages from the culture medium. Using this new protocol would likely have allowed us to obtain higher infection rates. However, this does not invalidate our comparative results because our protocol simply uses fewer infectious spores, but does not affect the cells’ susceptibility to infection; therefore, the observed differences remain valid.

Regarding the scope of our study, future work could benefit from testing a broader range of strains for each *Encephalitozoon* sp. While the genetic diversity of *E. intestinalis* is considered to be low [21], *E. hellem* [15, 35] and *E. cuniculi* [6, 29, 32] encompass several genotypes. Expanding the analysis to different strains or genotypes within the same species would further strengthen and refine our findings, particularly in light of previously reported inter-strain variability for *E. hellem* [14] or *Anncaliia algerae* [18].

Another avenue for improvement would be the use of stem cell-derived systems, such as differentiated 2D cultures or 3D organoids, which more accurately replicate the architecture and functions of host tissues, thereby providing more relevant *in vitro* models for studying host-pathogen interactions [19]. In the case of microsporidia, these approaches could better reflect the natural cellular niches and physiological conditions necessary for their development, while potentially improving culture efficiency and growth robustness. Research on intestinal organoids and stem cell-derived models has already demonstrated its value for the study of intracellular pathogens [36].

## Conclusions

In conclusion, our results show significant variability in the infection efficiency of microsporidia from the genus *Encephalitozoon*, not only between species (*i.e.*, *E. intestinalis*, *E. hellem*, and *E. cuniculi*) but also according to cell lines. In our experimental conditions, TC7 cells were the most susceptible to infection. These data provide valuable information on the specific interactions between microsporidia and host cells, and offer important guidance for selecting the most appropriate *in vitro* model. This choice could have a crucial impact on experimental results. Therefore, our results also suggest that it would be advisable to validate experimental data on at least two distinct cell lines to strengthen the robustness of the results and eliminate the “model” effect.

## Supplementary material

*Supplementary Table S1*. Raw data for calculation of infection rates of each *Encephalitozoon* species based on cell lines and biological replicate. The total number of cells on the day of infection and 48 hours later is taken into account when calculating the infection rate. The infection rate is calculated by dividing the total number of infected cells at 48 hours post-infection by the number of viable cells at the time of infection. PI: post-infection; p25: 25^th^ percentile; p75: 75^th^ percentile; SD: standard deviation. *Supplementary Table S2*. *P*-values for pairwise comparisons of infection rates between the six cell lines for each of the three *Encephalitozoon* species. Regarding *E. hellem*, the overall *p*-value was not significant (*p* = 0.08), so pairwise comparisons were not carried out. Significant *p*-values (≤ 0.05) are shown in bold. *Supplementary Table S3*. *P*-values for pairwise comparisons of the surface area of infectious foci between the six cell lines for each of the three *Encephalitozoon* species. Significant *p*-values (≤ 0.05) are shown in bold. The supplementary material of this article is available at https://www.parasite-journal.org/10.1051/parasite/2026026/olm.
