# Supplementary material for: Influence of host cell line and microsporidian species in the in vitro infection efficiency of Encephalitozoon spp
Source: Parasite. 2026 Apr 23;33:27. doi: 10.1051/parasite/2026026 (PMC13107947; doi:10.1051/parasite/2026026)
Supplement: Supplementary file 2 — Supplementary Table S2. P-values for pairwise comparisons of infection rates between the six cell lines for each of the three Encephalitozoon species. [file parasite-33-27-s2.pdf]

Supplementary Table S2. *P*-values for pairwise comparisons of infection rates between the six cell lines for each of the three *Encephalitozoon* species.

Regarding *E. hellem*, the overall *p*-value was not significant ($p = 0.08$), so pairwise comparisons were not carried out. Significant *p*-values (≤ 0.05) are shown in bold.

E. intestinalis	**Vero**	**MRC-5**	**TC7**	**T84**	**HT-29**	**HCT 116**
**Vero**						
**MRC-5**	0.76					
**TC7**	0.98	0.24				
**T84**	1.00	0.81	0.96			
**HT-29**	0.14	0.93	**0.01**	0.17		
**HCT 116**	0.40	1.00	0.07	0.45	1.00	

E. cuniculi	**Vero**	**MRC-5**	**TC7**	**T84**	**HT-29**	**HCT 116**
**Vero**						
**MRC-5**	0.98					
**TC7**	1.00	0.95				
**T84**	1.00	0.99	1.00			
**HT-29**	0.18	0.68	0.13	0.30		
**HCT 116**	0.18	0.68	0.13	0.30	1.00	
