# Supplementary material for: Influence of host cell line and microsporidian species in the in vitro infection efficiency of Encephalitozoon spp
Source: Parasite. 2026 Apr 23;33:27. doi: 10.1051/parasite/2026026 (PMC13107947; doi:10.1051/parasite/2026026)
Supplement: Supplementary file 3 — Supplementary Table S3. P-values for pairwise comparisons of the surface area of infectious foci between the six cell lines for each of the three Encephalitozoon species. [file parasite-33-27-s3.pdf]

Supplementary Table S3. *P*-values for pairwise comparisons of the surface area of infectious foci between the six cell lines for each of the three *Encephalitozoon* species.

Significant *p*-values (≤ 0.05) are shown in bold.

E. intestinalis	**Vero**	**MRC-5**	**TC7**	**T84**	**HT-29**	**HCT 116**
**Vero**						
**MRC-5**	**<0.001**					
**TC7**	**<0.001**	1.00				
**T84**	**0.003**	0.91	0.81			
**HT-29**	**0.02**	0.49	0.35	1.00		
**HCT 116**	0.21	0.10	0.06	0.98	1.00	

E. hellem	**Vero**	**MRC-5**	**TC7**	**T84**	**HT-29**	**HCT 116**
**Vero**						
**MRC-5**	1.00					
**TC7**	0.30	0.91				
**T84**	1.00	1.00	0.79			
**HT-29**	**<0.001**	**<0.001**	**0.004**	**<0.001**		
**HCT 116**	0.20	0.77	1.00	0.62	**0.03**	

E. cuniculi	**Vero**	**MRC-5**	**TC7**	**T84**	**HT-29**	**HCT 116**
**Vero**						
**MRC-5**	**<0.001**					
**TC7**	0.99	**<0.001**				
**T84**	0.98	**0.001**	1.00			
**HT-29**	0.99	**<0.001**	0.37	0.31		
**HCT 116**	0.93	**<0.001**	0.22	0.18	1.00	
